# Safety of COVID-19 Vaccine in Patients with Cancer in a High-Volume Comprehensive Cancer Center

**DOI:** 10.1093/oncolo/oyab037

**Published:** 2022-02-11

**Authors:** Antonella Brunello, Valentina Guarneri, Marina Coppola, Matteo Bernardi, Ketti Ottolitri, Maria Grazia Ghi, Eleonora Mioranza, Federica Vianello, Michele Gottardi, Sara Lonardi, Vittorina Zagonel

**Affiliations:** 1 Medical Oncology 1, Veneto Institute of Oncology IOV – IRCCS, Padua, Italy; 2 Department of Surgery, Oncology and Gastroenterology, University of Padova, Padova, Italy; 3 Pharmacy, Veneto Institute of Oncology IOV – IRCCS, Padua, Italy; 4 Healthcare Professions, Veneto Institute of Oncology IOV – IRCCS, Padua, Italy; 5 Risk Management, Veneto Institute of Oncology IOV – IRCCS, Padua, Italy; 6 Medical Oncology 2, Veneto Institute of Oncology IOV – IRCCS, Padua, Italy; 7 Radiotherapy, Veneto Institute of Oncology IOV – IRCCS, Padua, Italy; 8 Onco Hematology, Veneto Institute of Oncology IOV – IRCCS, Castelfranco Veneto, Italy; 9 Medical Oncology 3, Veneto Institute of Oncology IOV – IRCCS, Castelfranco Veneto, Italy

**Keywords:** cancer, chemotherapy, mRNA, vaccine, COVID-19

## Abstract

**Background:**

Few data are available on the safety of COVID-19 vaccines in cancer patients undergoing active cancer-directed treatment.

**Patients and Methods:**

This case series analyzes outcomes in terms of adverse events in 5297 patients undergoing anti-cancer treatment who were vaccinated with anti-SARS-CoV-2 Pfizer-BioNTech vaccine at a single cancer center from March 6, 2021 to May 9, 2021. Adverse events were retrieved from the national Italian pharmacovigilance platform (http://www.vigicovid.it).

**Results:**

Of the 5297 patients treated for either solid tumors (87%) or onco-hematologic malignancies (13%) who were vaccinated, 8 adverse drug reactions (ADRs) were reported. One was a severe ADR and 7 were non-severe ADRs. Non-severe ADRs resolved within 48 hours.

**Conclusion:**

BNT162b2 Pfizer-BioNTech vaccine was safely administered in the largest cohort of cancer patients reported to date.

## Teaser

Despite being highly recommended, the efficacy and safety of COVID-19 vaccines in patients with cancer is unknown. This article reports results with the Pfizer-BioNTech vaccine in a series of cancer patients.

## Introduction

Morbidity and mortality from COVID-19 in patients with cancer undergoing cancer-directed treatment are high, with approximately one-third of patients experiencing severe course of disease, approximately three quarters requiring hospitalization, with mortality rates of approximately 30%.^1,2^ Patients with hematologic cancer have been shown to have higher mortality compared with patients with solid cancer.^1-3^

The risk of a severe form of COVID-19 is mainly related to comorbidity, with chemotherapy within the last 30 days before COVID-19 diagnosis being associated with higher risk of death.^1,4^

COVID-19 is also associated with delay in treatment with consequent potential impact on oncologic outcomes, in light of prioritization policies with postponement of chemotherapy, radiotherapy, and/or surgery, as well as limited access to supportive care that impact quality of life.^5^

Although type and degree of immunosuppression can vary substantially among heterogeneous patient groups, there is mounting evidence that patients with cancer do not develop as much humoral immunity as non-cancer subjects and may remain contagious and able to spread SARS CoV2 for several months.^6^ Yet, recent data suggest a role of CD8+T-cell response that might provide protection in patients with cancer even in the setting of defective humoral immunity.^7^

COVID-19 vaccines have been made available since the end of 2020. There are currently 2 mRNA vaccines (Pfizer-BioNTech and Moderna) and 2 adenovirus-based vaccines (Oxford-AstraZeneca and Johnson & Johnson) for which phase III placebo-controlled randomized clinical trials show an impressive efficacy.

Based on their high efficacy as per results of phase III trials,^8,9^ mRNA vaccines have been recommended for use in immunocompromised and frail patients by national and international guidelines.^10,11^

Despite being highly recommended, the efficacy and safety profiles of vaccines against SARS-CoV-2 in patients with cancer are unknown since they were excluded by registration trials. Indeed, at the ESMO 2021 congress new data on efficacy of COVID-19 vaccines in patients with cancer have been presented, alongside with preliminary safety data, which hinted at a safety profile in the patient with cancer population which could overlap to the that observed in the general population.^11-15^

As per the Veneto Oncological Network (Rete Oncologica Veneta, ROV) criteria, Oncology Units in the Veneto Region are in charge for the vaccination of patients who either are being treated with chemotherapy, immunotherapy, and targeted therapy, or have completed the treatment in the past 6 months.

## Results

From March 6 to May 9 2021, a total of 5297 patients treated at Veneto Institute of Oncology, IOV for either solid tumors (87%) or onco-hematologic malignancies (13%) have been vaccinated with two 30 μg doses of the BNT162b2 Pfizer-BioNTech COVID-19 vaccine (Comirnaty©) administered intramuscularly 21 days apart.

Among all patients with the above stated criteria who were therefore proposed vaccination, 207 (3.9%) refused to get vaccinated. Overall 10.820 doses of vaccines have been administered, with 226 patients (1.8%) who had received the first dose not completing the planned second dose because of either worsening of general conditions, admission to other hospital wards, occurrence of COVID-19 infection, or death. Indeed, a minority of patients (14 patients) developed COVID-19 infection right after first dose.

Overall 4324 patients (81.6%) were actively receiving oncological treatment, and 973 (18.4%) had completed it in the past 6 months. Details on treatment are presented in Table 1.

**Table 1.** Oncological treatment details for patients on-treatment at the time of COVID-19 vaccination (*N* = 4324).

	*N*	%
Cytotoxic chemotherapy	1669	38.6
Immunotherapy	341	7.9
Targeted therapy	934	21.6
Endocrine therapy	615	14.2
Chemotherapy + immunotherapy	85	2.0
Targeted therapy + endocrine therapy	364	8.4
Immunotherapy + targeted therapy	68	1.6
Chemotherapy + targeted therapy	248	5.7

No specific timing regarding the chemotherapy schedule was required, except patients were not vaccinated on the same day of chemotherapy. As a general approach, although the first vaccine dose was preferentially administered before starting cytotoxic chemotherapy whenever possible.

Patients remained under observation for 15 or 30 minutes after vaccination according to medical history. Overall, out of 8 adverse drug reactions (ADRs) reported through the national pharmacovigilance website (www.vigicovid.it), 1 was a severe ADR and 7 were non-severe ADRs. The severe ADR was a central retinal artery thrombosis in a patient undergoing treatment with gefitinib for EGFR-mutated non–small cell lung cancer, causing blindness in the affected eye. Patient was given anti-platelet agent acetil-salicilic acid and is being followed up with condition not resolved to date. All the reported non-severe ADRs (“fainting, hypertensive episode, hypotension”; “skeletal pain, muscle pain, fever, rhinitis”; “back pain, fatigue, fever; reddening at injection site, hot flashes”; “tongue pruritus”; “paresthesia, pruritus, skin rash”; “headache, myalgia, fever, rigidity”, respectively) resolved in 48 hours. Patients were observed for severe adverse reaction up to 1 month after second dose, with all the reported ADRs developing within 14 days from the vaccine administration.

Only 4 patients showed symptoms consistent with hypersensitivity reactions, which were not serious and resolved in <1 hour; no patients required hospitalization for hypersensitivity reactions, and they resolved with medical management with anti-histamine drugs in 3 cases, and with observation in the other case.

## Discussion

Our data show that COVID-19 vaccination with BNT162b2 Pfizer-BioNTech vaccine was highly safe in one of the largest reported series of patients with cancer treated with chemotherapy, immunotherapy, or targeted therapy reported to date.

The rate of vaccine refusal in our cohort of cancer patients undergoing cancer-directed treatment was lower than expected. Indeed, colleagues of the Regina Elena Cancer Institute- Rome reported a 11% refusal rate in their cohort of 914 patients, with refusal being mostly due to fear of adverse events.^16^

These findings are consistent with the reassuring safety signals regarding the BNT162b2 mRNA COVID-19 vaccine reported in smaller cohorts of patients with cancer.^11-15,17^ In our case series the only severe adverse event recorded was a central retinal artery occlusion, which notwithstanding the unsure causality relationship, negatively compares with findings from general population in which COVID mRNA vaccination was shown to be protective against cerebrovascular events.^18^

As for the scheduling, an every-3 week dosing schedule was feasible, with only 1.8% of patients not completing the full schedule with the second dose. Indeed, recent data from the UK showed poor efficacy of a single dose of the BNT162b2 vaccine in patients with cancer, whereas immunogenicity increased significantly in patients with solid cancer within 2 weeks of a vaccine boost at day 21 after the first dose.^17^ Recent data also point at the usefulness of an additional dose of mRNA vaccine.^19^

## Conclusion

These data confirm the safety of the BNT162b2 Pfizer-BioNTech vaccine in the largest cohort of cancer patients reported to date.

As for the effectiveness, longer follow-up is needed in order to provide data on the rate of COVID19 infections among fully vaccinated cancer patients.

## References

[1] Lièvre A , Turpin A, Ray-Coquard I, et al Risk factors for Coronavirus Disease 2019 (COVID-19) severity and mortality among solid cancer patients and impact of the disease on anticancer treatment: a French nationwide cohort study (GCO-002 CACOVID-19). Eur J Cancer. 2020;141:62-81. 33129039 10.1016/j.ejca.2020.09.035 PMC7543792

[2] Guarneri V , Bassan F, Zagonel V, et al Epidemiology and clinical course of severe acute respiratory syndrome coronavirus 2 infection in cancer patients in the Veneto Oncology Network: the Rete Oncologica Veneta covID19 study. Eur J Cancer. 2021;147:120-127. 33647547 10.1016/j.ejca.2021.01.021 PMC7857033

[3] Passamonti F , Cattaneo C, Arcaini L, et al Clinical characteristics and risk factors associated with COVID-19 severity in patients with haematological malignancies in Italy: a retrospective, multicentre, cohort study. Lancet Haematol. 2020;7(10):e737-e745. 10.1016/S2352-3026(20)30251-9 32798473 PMC7426107

[4] Yekedüz E , Utkan G, Ürün Y. A systematic review and metaanalysis: the effect of active cancer treatment on severity of COVID-19. Eur J Cancer. 2020;141:92-1 04. 33130550 10.1016/j.ejca.2020.09.028 PMC7538140

[5] Riera R , Bagattini ÂM, Pacheco RL, et al Delays and disruptions in cancer health care due to COVID-19 pandemic: systematic review. JCO Glob Oncol. 2021;7:311-323. 33617304 10.1200/GO.20.00639 PMC8081532

[6] Solodky ML , Galvez C, Russias B, et al Lower detection rates of SARS-COV2 antibodies in cancer patients versus health care workers after symptomatic COVID-19. Ann Oncol. 2020;31(8):1087-1 088. 32360743 10.1016/j.annonc.2020.04.475 PMC7252166

[7] Bange EM , Han NA, Wileyto P, et al CD8+T cells contribute to survival in patients with COVID-19 and hematologic cancer. Nat Med. 2021. 10.1038/s41591-021-01386-7 PMC8291091 34017137

[8] Polack FP , Thomas SJ, Kitchin N, et al Safety and efficacy of the BNT162b2 mRNA Covid-19 vaccine. N Engl J Med. 2020;383(27):2603-2615. 33301246 10.1056/NEJMoa2034577 PMC7745181

[9] Baden LR , El Sahly HM, Essink B, et al Efficacy and safety of the mRNA-1273 SARS-CoV-2 vaccine. N Engl J Med. 2021;384(5):403-416. 33378609 10.1056/NEJMoa2035389 PMC7787219

[10] Silvestris N , Di Maio M, Russo A, et al COVID-19 infection in cancer patients: what has been the contribution of Associazione Italiana Oncologia Medica (AIOM) to oncological care since the beginning of the first pandemic wave? ESMO Open. 2021;6(2):100100. 10.1016/j.esmoop.2021.100100 33819751 PMC7973080

[11] ASCO (American Society of Clinical Oncology) Coronavirus Resources. Accessed January 25, 2022. https://www.asco.org/covid-resources

[12] Thomas SJ , Perez JL, Lockhart SP, et al Subgroup analysis of efficacy/safety from a global phase III randomized trial of the BNT162b2 (tozinameran) mRNA vaccine. Ann Oncol. 2021;32(Suppl_5):S1129-S1163. 10.1016/annonc/annonc713

[13] Zemel M , Kian W, Kestenbaum EH, et al. Safety of the BNT162b2 mRNA COVID-19 vaccine in oncologic patients undergoing numerous cancer treatment options. Ann Oncol. 2021;32(suppl_5):S1129-S1163. 10.1016/annonc/annonc713 PMC8758044 35029223

[14] Obermannova R , Demlova R, Selingerova I, et al CoVigi phase IV multicentric trial evaluating COVID-19 vaccination adverse events and immune response dynamics in cancer patients: first results on antibody and cellular immunity. Ann Oncol. 2021;32(Suppl_5):S1129-S1163. 10.1016/annonc/annonc713

[15] Waldhorn I , Ben-Aharon I, Holland R, et al Efficacy and toxicity of BNT162b2 vaccine in cancer patients. Ann Oncol. 2021;32(Suppl_5):S1129-S1163. 10.1016/annonc/annonc713

[16] Di Noia V , Renna D, Barberi V, et al The first report on Covid-19 vaccine refusal by cancer patients in Italy: early data from a single-institute survey. Eur J Cancer. 2021;153:260-264. 10.1016/j.ejca.2021.05.006 34183225 PMC8149194

[17] Monin L , Laing AG, Muñoz-Ruiz M, et al Safety and immunogenicity of one versus two doses of the COVID-19 vaccine BNT162b2 for patients with cancer: interim analysis of a prospective observational study. Lancet Oncol. 2021; 22(6):765-778. 33930323 10.1016/S1470-2045(21)00213-8 PMC8078907

[18] Barda N , Dagan N, Ben-Shlomo Y, et al Safety of the BNT162b2 mRNA Covid-19 vaccine in a nationwide setting. N Engl J Med. 2021;385(12):1078–1090. 34432976 10.1056/NEJMoa2110475 PMC8427535

[19] Shroff RT , Chalasani P, Ran Wei R, et al Immune responses to two and three doses of the BNT162b2 mRNA vaccine in adults with solid tumors. Nat Med. 2021;27(11):2002-2011. 10.1038/s41591-021-01542-z. 34594036 PMC9004706

